# RNA sequencing data for gamma radiation response in the extremotolerant tardigrade *Ramazzottius varieornatus*

**DOI:** 10.1016/j.dib.2021.107111

**Published:** 2021-05-09

**Authors:** Yuki Yoshida, Daiki D. Horikawa, Tetsuya Sakashita, Yuichiro Yokota, Yasuhiko Kobayashi, Masaru Tomita, Kazuharu Arakawa

**Affiliations:** a Keio University, Institute for Advanced Biosciences, Tsuruoka, Yamagata 997-0035, Japan; b Keio University, Graduate School of Media and Governance, Systems Biology Program, Fujisawa, Kanagawa 252-0882, Japan; c National Institutes for Quantum and Radiological Science and Technology, Takasaki Advanced Radiation Research Institute, Takasaki, Gunma 370-1292, Japan; d Keio University, Faculty of Environment and Information Studies, Fujisawa, Kanagawa 252-0882, Japan

**Keywords:** *Ramazzottius varieornatus*, Extremotolerant, Transcriptome sequencing, Gamma ray

## Abstract

Tardigrades are microscopic animals of which terrestrial species are capable of tolerating extreme environments by entering a desiccated ametabolic state known as anhydrobiosis. Intriguingly, they survive high dosage gamma rays (>4,000 Gy), possibly through a mechanism known as cross-tolerance. We hypothesized that anhydrobiosis genes are also regulated during cross-tolerance, thus we submitted *Ramazzottius varieornatus* to 500 Gy ^60^Co gamma-ray and conducted time-course low-input RNA-Seq. The gene expression was quantified with RSEM and differential expression was determined with DEseq2. Differentially expressed genes were submitted to gene ontology enrichment analysis with GOStat. The transcriptome dynamically shifted nine hours post-exposure.

**Specifications Table**

**Table:** 

Subject	Biological sciences
Specific subject area	Omics: Transcriptomics
Type of data	Table Figure
How data were acquired	Total RNA was extracted from approximately 30 individuals per sample with Direct-zol RNA Miniprep and an Illumina sequencing library was constructed with NEBNext Ultra RNA Library Prep Kit for Illumina. The final 24 libraries were pooled and was sequenced on the Illumina NextSeq 500 platform.
Data format	Raw: raw reads (FASTQ) Analysed: RNA-seq data files (counts and DEG lists)
Parameters for data collection	Total RNAs were extracted from 30 individuals of *Ramazzottius varieornatus* in a time-course between 24 hours after exposure to 500 Gy of ^60^Co gamma ray.
Description of data collection	RNA-Seq data was sequenced on a NextSeq 500 platform. Raw reads were mapped to the *Ramazzottius varieornatus* coding sequences (v101) with bowtie2 and quantified with RSEM. Mapped counts were tested with DESeq2 and genes with FDR <0.05 were designated as differentially expressed genes.
Data source location	Institution: Institute for Advanced Biosciences, Keio University City/Town/Region: Tsuruoka, Yamagata Country: Japan
Data accessibility	Repository name: NCBI GEO Data identification number: GSE166661 Direct URL to data: https://www.ncbi.nlm.nih.gov/geo/query/acc.cgi?acc=GSE166661 Repository name: FigShare Data identification number: Direct URL to data: https://figshare.com/articles/dataset/RNA_sequencing_data_for_gamma_radiation_response_in_the_extremotolerant_tardigrade_Ramazzottius_varieornatus/14343788
Related research article	None

## Value of the Data

• These data are the first preliminary data of valuable time-course transcriptome sequence data of the extremotolerant tardigrade, *Ramazzottius varieornatus*, exposed to a high dosage of ^60^Co gamma-ray (500 Gy), providing the community a basis for studying the gamma-ray response in tardigrades.
• These data provide researchers new insights about molecular processes affected after gamma-ray exposure in tardigrades and the expression of genes with unknown functions.
• The data shown here would be a basis of understanding the anti-oxidative stress response in tardigrades, leading to the identification of anhydrobiosis related factors.

## 1 Data Description

**Fig. 1:**
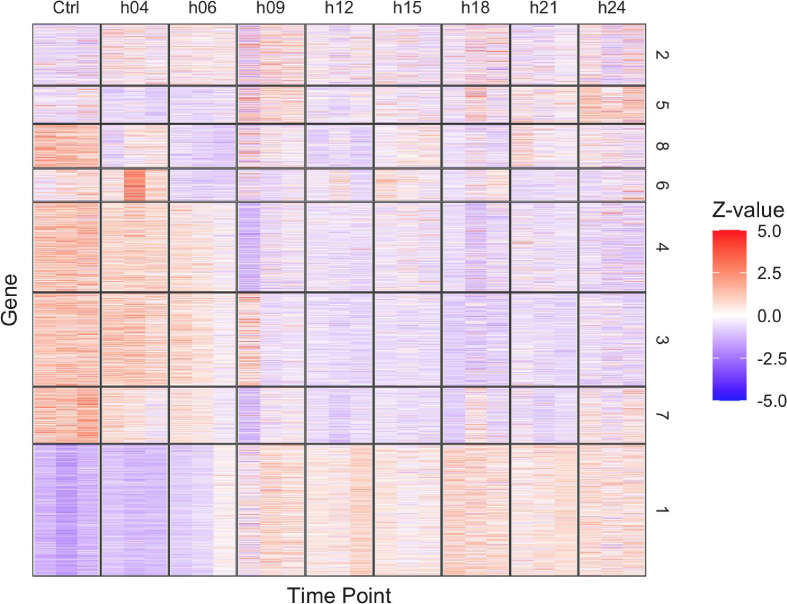
Expression profile of gamma ray response.

This manuscript presents a time-course transcriptomic dataset obtained from the gamma-ray exposure-response of the extremotolerant tardigrade, *Ramazzottius varieornatus*. This RNA-Seq data employed low-input RNA-Seq, 30 ng of total RNA extracted from 30 individuals per sample. Table 1 displays all RNA-Seq data obtained in this study. The gene expression was quantified with RSEM based on the bowtie2 mapping against the coding sequences of *R. varieornatus* (Table S1), and a total of 4,138 genes was determined differentially expressed by DESeq2 (FDR <0.05, Figs. 1, S1, Tables S1–S3). Gene ontology enrichment analysis of between all-time points illustrates the transcriptomic response against the extensive stress caused by the gamma-ray exposure (Table S4). Using this data requires caution, as sampling was conducted in technical triplicates and may show less variance that may be seen between biological replicates.

**Table 1:** Summary of RNA-Seq data and mapping ratio to coding sequences and whole genome.

Sample	#reads	#reads mapped to CDS	ratio	#reads mapped to genome	ratio
Ctrl-1	27,303,730	14,826,259	54.3%	25,416,130	93.1%
Ctrl-2	18,863,864	10,580,613	56.1%	17,689,807	93.8%
Ctrl-3	10,795,407	5,213,237	48.3%	9,685,301	89.7%
h04-1	31,453,873	19,645,842	62.5%	29,682,118	94.4%
h04-2	32,246,798	19,862,228	61.6%	30,392,041	94.2%
h04-3	20,411,600	12,349,871	60.5%	19,351,873	94.8%
h06-1	30,352,146	19,447,215	64.1%	28,583,770	94.2%
h06-2	28,301,579	18,664,499	65.9%	26,789,528	94.7%
h06-3	22,361,535	13,332,811	59.6%	20,592,696	92.1%
h09-1	17,863,042	9,165,576	51.3%	14,412,898	80.7%
h09-2	4,908,154	3,161,182	64.4%	4,643,555	94.6%
h09-3	25,774,045	16,828,096	65.3%	24,305,163	94.3%
h12-1	15,646,686	10,168,892	65.0%	14,505,394	92.7%
h12-2	16,376,288	10,652,046	65.0%	15,524,500	94.8%
h12-3	7,603,714	5,126,152	67.4%	7,123,186	93.7%
h15-1	11,404,989	7,644,237	67.0%	10,797,238	94.7%
h15-2	5,350,675	3,135,583	58.6%	5,085,119	95.0%
h15-3	8,434,690	4,136,376	49.0%	7,357,727	87.2%
h18-1	26,921,173	17,248,331	64.1%	25,722,001	95.5%
h18-2	13,173,806	7,891,669	59.9%	12,474,527	94.7%
h18-3	10,338,694	5,490,802	53.1%	9,735,742	94.2%
h21-1	12,790,261	5,043,921	39.4%	11,930,133	93.3%
h21-2	12,981,419	5,592,580	43.1%	12,313,889	94.9%
h21-3	25,342,537	13,651,405	53.9%	23,989,463	94.7%
h24-1	4,193,581	2,612,440	62.3%	4,012,806	95.7%
h24-2	5,020,493	2,369,600	47.2%	4,700,011	93.6%
h24-3	3,461,282	1,465,710	42.3%	3,218,528	93.0%

TMM-normalized TPM values of differentially expressed genes were Z-scaled and visualized as a heat map. The spearman correlation between each gene were clustered into 8 clusters with the Ward method, indicated by the numbers on the right end of the plot. Ctrl: Non-irradiated controls, h04-h24: samples from 4h-24h post exposure.

## 2 Experimental Design, Materials and Methods

### 2.1 Low-input time-course RNA-Seq

Tardigrades were reared based on the method established by our previous study [1]. *R. varieornatus* were placed on 90 mm plastic plates layered with 2% volvic agar at 22 °C and fed with *Chlorella vulgaris*. Culture plates were transported to Japan Atomic Energy Agency (JAEA), Takasaki Advanced Radiation Research Institute, and were incubated at 22˚C for more than 12 hours to eliminate the effects of the transportation. 2,000 tardigrades were suspended in two 1.5 mL tubes and filled with 100 µL of distilled water. These tubes were then irradiated with 500 Gy of ^60^Co gamma ray, at a dose rate of 2,008 Gy/hour at a distance of 63 cm from the ^60^Co source. The content of each tubes was spread over a 90 mm agar layered plate and incubated at 22 °C until further sampling. At each sampling point (4h, 6h. 9h, 12h, 15h, 18h, 24h), 30 animals were randomly sampled with 10 µL Pipettes with the least water as possible, and placed into 200 µL tubes containing 150 µL of TRIzol (Life Technologies, 3 technical replicates per condition), and were preserved at −20 °C.

Total RNA was extracted with Direct-Zol (Zymo Research) and 20 ng of total RNA was submitted to cDNA synthesis, sequencing adaptor and index ligation, PCR amplification using NEBNext Ultra RNA Kit (New England BioLabs Japan) according to manufacture protocol. RNA was extracted at least within 1 week of irradiation. The cDNA was amplified by at least 15 cycles of PCR (h1-1~h6-2: 15 cycles, h6-3~h21-3: 21 cycles, h24-1~h24-3: 23 cycles). The quality of the nucleic acids was validated with Nanodrop (Thermo Scientific) and quantified by Qubit RNA High Sensitivity or dsDNA Broad Range (Life Technologies). The library length distribution was validated by Tapestation D1000 (Agilent). Since sample h09_1 showed signs of adapter dimers, the pooled samples were size selected for the main band. Sequencing was performed with NextSeq 500 (Illumina) following manufacturer's instructions, using NextSeq 500 High Output Kit (75bp single end). Raw reads were de-multiplexed by bcl2fastq v2.15.0.4 (Illumina) and each sequencing lane were merged. Each sample were validated with FastQC v0.11.2 [2].

### 2.2 Informatics analysis

Gene expression was quantified with RSEM using the align_and_estimate_abundance.pl utility from the Trinity suite v2.11.0 [3] using the *R. varieornatus* coding sequence [4]. Reads were also mapped to the genome with bwa MEM v0.7.12-r1039. The raw mapped counts were tested for differential expression with DESeq2 v1.22.2 [5] and genes with FDR <0.05 and fold change above 2 were called as differentially expressed genes. Gene expression profiles were clustered by Ward method based on the spearman's correlation and was visualized as a heatmap with ggplot2. A gene ontology enrichment analysis was performed for each time point with GOstat v2.48.0 [6], and terms with a single observation was excluded. G-language Genome Analysis Environment v1.9.1 [7,8] were used for sequence manipulation and data parsing.

## Ethics Statement

All experiments were conducted following the Japanese law and guidelines from The Science Council of Japan, as well as the Ministry of Education, Culture, Sports, Science and Technology (MEXT) of Japan.

## CRediT Author Statement

**Yuki Yoshida:** Conceptualization, Data curation, Formal analysis, investigation, Methodology, Validation, Visualization, Writing - original draft, Writing - review & editing; **Daiki D. Horikawa:** Methodology; **Tetsuya Sakashita:** Methodology; **Yuichiro Yokota:** Methodology; **Yasuhiko Kobayashi:** Supervision; **Masaru Tomita:** Supervision, Resources; **Kazuharu Arakawa:** Conceptualization, Funding acquisition, Project administration, Resources, Writing - review & editing.

## Declaration of Competing Interest

The authors declare that they have no known competing financial interests or personal relationships which have or could be perceived to have influenced the work reported in this article.

## Appendix Supplementary materials

Image, application 1

Image, application 2

## Footnotes

Supplementary material associated with this article can be found in the online version at doi:10.1016/j.dib.2021.107111.
