# Supplementary figures and images for: RNA sequencing data for gamma radiation response in the extremotolerant tardigrade Ramazzottius varieornatus
Source: Data Brief. 2021 May 9;36:107111. doi: 10.1016/j.dib.2021.107111 (PMC8166744; doi:10.1016/j.dib.2021.107111)

# Cluster Dendrogram

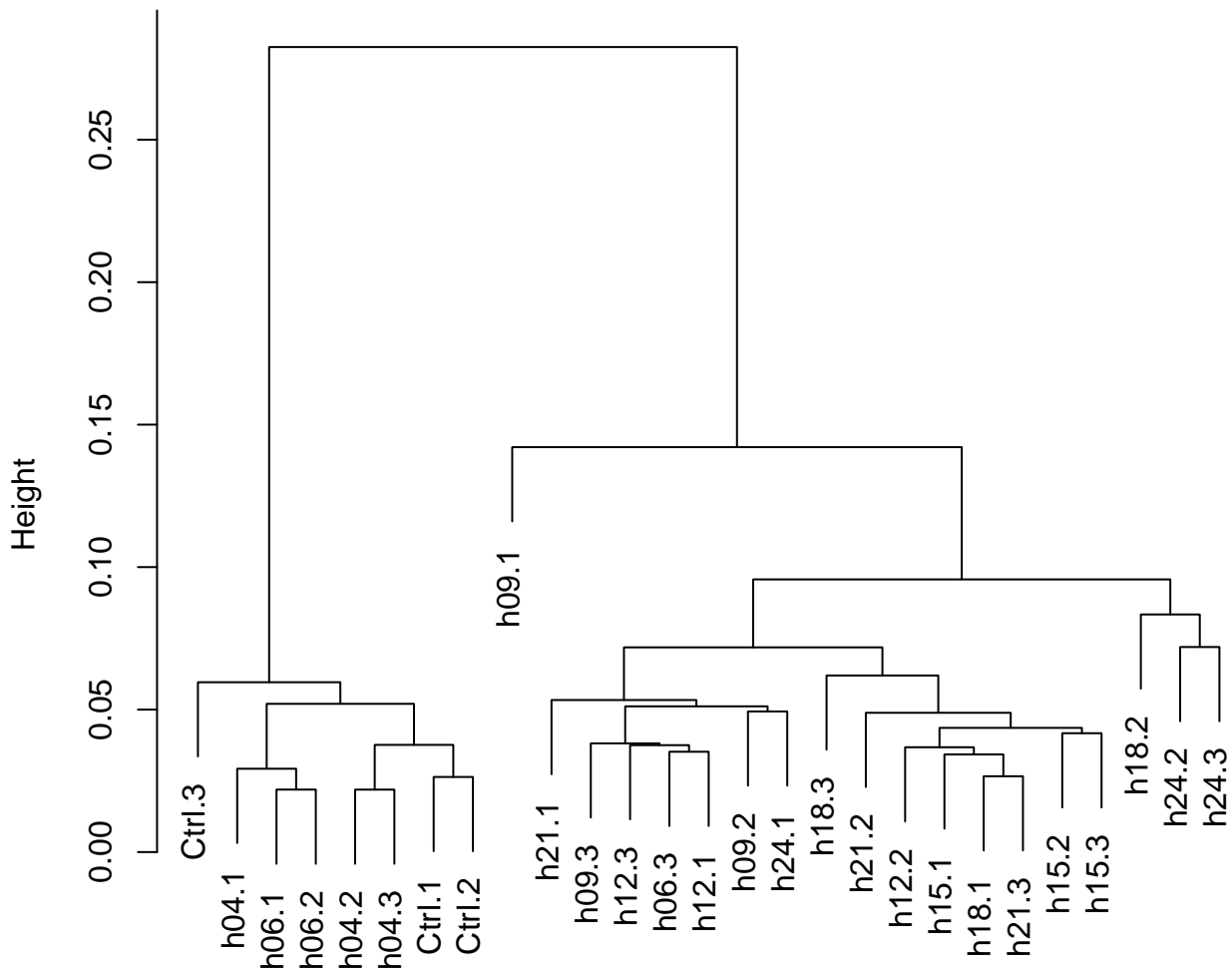

as.dist(1 - cor(data, method = "spearman"))  
hclust (\*, "ward.D")

Supplement: Supplementary file 1 [file mmc1.pdf]
