# Supplementary material for: RNA sequencing data for gamma radiation response in the extremotolerant tardigrade Ramazzottius varieornatus
Source: Data Brief. 2021 May 9;36:107111. doi: 10.1016/j.dib.2021.107111 (PMC8166744; doi:10.1016/j.dib.2021.107111)
Supplement: Supplementary file 2 [file mmc2.zip › mmc2/S2352340921003954.zip!/DIB Article Template 3.2.1-REVISE2-clean.docx]

**Article Title**

**RNA sequencing data for gamma radiation response in the extremotolerant tardigrade *Ramazzottius varieornatus***

**Authors**

Yuki Yoshida ^1,2^, Daiki D. Horikawa^1,2^, Tetsuya Sakashita^3^, Yuichiro Yokota^3^, Yasuhiko Kobayashi^3^, Masaru Tomita^1,2,4^, Kazuharu Arakawa^1,2,4^

**Affiliations**

1. Keio University, Institute for Advanced Biosciences, Tsuruoka, Yamagata, 997-0035, Japan.
2. Keio University, Graduate School of Media and Governance, Systems Biology Program, Fujisawa, Kanagawa, 252-0882, Japan.
3. National Institutes for Quantum and Radiological Science and Technology, Takasaki Advanced Radiation Research Institute, Takasaki, Gunma, 370-1292, Japan.
4. Keio University, Faculty of Environment and Information Studies, Fujisawa, Kanagawa, 252-0882, Japan.

**Corresponding author(s)**

Kazuharu Arakawa (gaou@sfc.keio.ac.jp), @gaou_ak

**Abstract**

Tardigrades are microscopic animals of which terrestrial species are capable of tolerating extreme environments by entering a desiccated ametabolic state known as anhydrobiosis. Intriguingly, they survive high dosage gamma rays (>4,000 Gy), possibly through a mechanism known as cross-tolerance. We hypothesized that anhydrobiosis genes are also regulated during cross-tolerance, thus we submitted *Ramazzottius varieornatus* to 500 Gy ^60^Co gamma-ray and conducted time-course low-input RNA-Seq. The gene expression was quantified with RSEM and differential expression was determined with DEseq2. Differentially expressed genes were submitted to gene ontology enrichment analysis with GOStat. The transcriptome dynamically shifted nine hours post-exposure.

**Keywords**

*Ramazzottius varieornatus*, extremotolerant, transcriptome sequencing, gamma ray

**Specifications Table**

| **Subject** | Biological sciences |
| --- | --- |
| **Specific subject area** | Omics: Transcriptomics |
| **Type of data** | Table  Figure |
| **How data were acquired** | Total RNA was extracted from approximately 30 individuals per sample with Direct-zol RNA Miniprep and an Illumina sequencing library was constructed with NEBNext Ultra RNA Library Prep Kit for Illumina. The final 24 libraries were pooled and was sequenced on the Illumina NextSeq 500 platform. |
| **Data format** | Raw: raw reads (FASTQ)  Analysed: RNA-seq data files (counts and DEG lists) |
| **Parameters for data collection** | Total RNAs were extracted from 30 individuals of *Ramazzottius varieornatus* in a time-course between 24 hours after exposure to 500 Gy of ^60^Co gamma ray. |
| **Description of data collection** | RNA-Seq data was sequenced on a NextSeq 500 platform. Raw reads were mapped to the *Ramazzottius varieornatus* coding sequences (v101) with bowtie2 and quantified with RSEM. Mapped counts were tested with DESeq2 and genes with FDR < 0.05 were designated as differentially expressed genes. |
| **Data source location** | Institution: Institute for Advanced Biosciences, Keio University  City/Town/Region: Tsuruoka, Yamagata  Country: Japan |
| **Data accessibility** | Repository name: NCBI GEO  Data identification number: GSE166661  Direct URL to data: https://www.ncbi.nlm.nih.gov/geo/query/acc.cgi?acc=GSE166661  Repository name: FigShare  Data identification number:  Direct URL to data: https://figshare.com/articles/dataset/RNA_sequencing_data_for_gamma_radiation_response_in_the_extremotolerant_tardigrade_Ramazzottius_varieornatus/14343788 |
| **Related research article** | **None** |

**Value of the Data**

1)	These data are the first preliminary data of valuable time-course transcriptome sequence data of the extremotolerant tardigrade, *Ramazzottius varieornatus*, exposed to a high dosage of ^60^Co gamma-ray (500 Gy), providing the community a basis for studying the gamma-ray response in tardigrades.

2)	These data provide researchers new insights about molecular processes affected after gamma-ray exposure in tardigrades and the expression of genes with unknown functions.

3)	The data shown here would be a basis of understanding the anti-oxidative stress response in tardigrades, leading to the identification of anhydrobiosis related factors.

**Data Description**

This manuscript presents a time-course transcriptomic dataset obtained from the gamma-ray exposure-response of the extremotolerant tardigrade, *Ramazzottius varieornatus*. This RNA-Seq data employed low-input RNA-Seq, 30 ng of total RNA extracted from 30 individuals per sample. Table 1 displays all RNA-Seq data obtained in this study. The gene expression was quantified with RSEM based on the bowtie2 mapping against the coding sequences of *R. varieornatus* (Table S1), and a total of 4,138 genes was determined differentially expressed by DESeq2 (FDR < 0.05, Figure 1, Figure S1, Table S1, Table S2, Table S3). Gene ontology enrichment analysis of between all-time points illustrates the transcriptomic response against the extensive stress caused by the gamma-ray exposure (Table S4). Using this data requires caution, as sampling was conducted in technical triplicates and may show less variance that may be seen between biological replicates.

Table 1: Summary of RNA-Seq data and mapping ratio to coding sequences and whole genome.

| Sample | #reads | #reads mapped to CDS | ratio | #reads mapped to genome | ratio |
| --- | --- | --- | --- | --- | --- |
| Ctrl-1 | 27,303,730 | 14,826,259 | 54.3% | 25,416,130 | 93.1% |
| Ctrl-2 | 18,863,864 | 10,580,613 | 56.1% | 17,689,807 | 93.8% |
| Ctrl-3 | 10,795,407 | 5,213,237 | 48.3% | 9,685,301 | 89.7% |
| h04-1 | 31,453,873 | 19,645,842 | 62.5% | 29,682,118 | 94.4% |
| h04-2 | 32,246,798 | 19,862,228 | 61.6% | 30,392,041 | 94.2% |
| h04-3 | 20,411,600 | 12,349,871 | 60.5% | 19,351,873 | 94.8% |
| h06-1 | 30,352,146 | 19,447,215 | 64.1% | 28,583,770 | 94.2% |
| h06-2 | 28,301,579 | 18,664,499 | 65.9% | 26,789,528 | 94.7% |
| h06-3 | 22,361,535 | 13,332,811 | 59.6% | 20,592,696 | 92.1% |
| h09-1 | 17,863,042 | 9,165,576 | 51.3% | 14,412,898 | 80.7% |
| h09-2 | 4,908,154 | 3,161,182 | 64.4% | 4,643,555 | 94.6% |
| h09-3 | 25,774,045 | 16,828,096 | 65.3% | 24,305,163 | 94.3% |
| h12-1 | 15,646,686 | 10,168,892 | 65.0% | 14,505,394 | 92.7% |
| h12-2 | 16,376,288 | 10,652,046 | 65.0% | 15,524,500 | 94.8% |
| h12-3 | 7,603,714 | 5,126,152 | 67.4% | 7,123,186 | 93.7% |
| h15-1 | 11,404,989 | 7,644,237 | 67.0% | 10,797,238 | 94.7% |
| h15-2 | 5,350,675 | 3,135,583 | 58.6% | 5,085,119 | 95.0% |
| h15-3 | 8,434,690 | 4,136,376 | 49.0% | 7,357,727 | 87.2% |
| h18-1 | 26,921,173 | 17,248,331 | 64.1% | 25,722,001 | 95.5% |
| h18-2 | 13,173,806 | 7,891,669 | 59.9% | 12,474,527 | 94.7% |
| h18-3 | 10,338,694 | 5,490,802 | 53.1% | 9,735,742 | 94.2% |
| h21-1 | 12,790,261 | 5,043,921 | 39.4% | 11,930,133 | 93.3% |
| h21-2 | 12,981,419 | 5,592,580 | 43.1% | 12,313,889 | 94.9% |
| h21-3 | 25,342,537 | 13,651,405 | 53.9% | 23,989,463 | 94.7% |
| h24-1 | 4,193,581 | 2,612,440 | 62.3% | 4,012,806 | 95.7% |
| h24-2 | 5,020,493 | 2,369,600 | 47.2% | 4,700,011 | 93.6% |
| h24-3 | 3,461,282 | 1,465,710 | 42.3% | 3,218,528 | 93.0% |

**Figure 1. Expression profile of gamma ray response.**

TMM-normalized TPM values of differentially expressed genes were Z-scaled and visualized as a heat map. The spearman correlation between each gene were clustered into 8 clusters with the Ward method, indicated by the numbers on the right end of the plot. Ctrl : Non-irradiated controls, h04-h24: samples from 4h-24h post exposure.

**Experimental Design, Materials and Methods**

**Low-input time-course RNA-Seq**

Tardigrades were reared based on the method established by our previous study [1]. *R. varieornatus* were placed on 90 mm plastic plates layered with 2% volvic agar at 22°C and fed with *Chlorella vulgaris*. Culture plates were transported to Japan Atomic Energy Agency (JAEA), Takasaki Advanced Radiation Research Institute, and were incubated at 22˚C for more than 12 hours to eliminate the effects of the transportation. 2,000 tardigrades were suspended in two 1.5 mL tubes and filled with 100 μL of distilled water. These tubes were then irradiated with 500 Gy of ^60^Co gamma ray, at a dose rate of 2,008 Gy/hour at a distance of 63 cm from the ^60^Co source. The content of each tubes was spread over a 90 mm agar layered plate and incubated at 22°C until further sampling. At each sampling point (4h, 6h. 9h, 12h, 15h, 18h, 24h), 30 animals were randomly sampled with 10 μL Pipettes with the least water as possible, and placed into 200 μL tubes containing 150 μL of TRIzol (Life Technologies, 3 technical replicates per condition), and were preserved at -20°C.

Total RNA was extracted with Direct-Zol (Zymo Research) and 20 ng of total RNA was submitted to cDNA synthesis, sequencing adaptor and index ligation, PCR amplification using NEBNext Ultra RNA Kit (New England BioLabs Japan) according to manufacture protocol. RNA was extracted at least within 1 week of irradiation. The cDNA was amplified by at least 15 cycles of PCR (h1-1~h6-2: 15 cycles, h6-3~h21-3: 21 cycles, h24-1~h24-3: 23 cycles). The quality of the nucleic acids was validated with Nanodrop (Thermo Scientific) and quantified by Qubit RNA High Sensitivity or dsDNA Broad Range (Life Technologies). The library length distribution was validated by Tapestation D1000 (Agilent). Since sample h09_1 showed signs of adapter dimers, the pooled samples were size selected for the main band. Sequencing was performed with NextSeq 500 (Illumina) following manufacturer’s instructions, using NextSeq 500 High Output Kit (75bp single end). Raw reads were de-multiplexed by bcl2fastq v2.15.0.4 (Illumina) and each sequencing lane were merged. Each sample were validated with FastQC v0.11.2 [2].

**Informatics analysis**

Gene expression was quantified with RSEM using the align_and_estimate_abundance.pl utility from the Trinity suite v2.11.0 [3] using the *R. varieornatus* coding sequence [4]. Reads were also mapped to the genome with bwa MEM v0.7.12-r1039. The raw mapped counts were tested for differential expression with DESeq2 v1.22.2 [5] and genes with FDR < 0.05 and fold change above 2 were called as differentially expressed genes. Gene expression profiles were clustered by Ward method based on the spearman’s correlation and was visualized as a heatmap with ggplot2. A gene ontology enrichment analysis was performed for each time point with GOstat v2.48.0 [6], and terms with a single observation was excluded. G-language Genome Analysis Environment v1.9.1 [7, 8] were used for sequence manipulation and data parsing.

**Supplementary files**

Figure S1: Sample dendrogram: The expression profiles of each transcript were clustered with the Ward method based on the spearman correlation and visualized as a dendrogram.

Table S1: Count table result from RNA-Seq mapping to CDS. Raw reads were mapped to the transcript coding sequences with RSEM and Bowtie2 and the number of mapped reads were counted. Transcript: Transcript ID, Ctrl: Control samples, h04-h24: samples from each corresponding time point, 1-3: replicate number.

Table S2: TMM-normalized TPM values. Raw reads were mapped to the transcript coding sequences with RSEM and Bowtie2 and the count table were normalized as TMM-corrected TPM values. Transcript: transcript ID, Ctrl: control samples, h04-h24: samples from each corresponding time point, 1-3: replicate number.

Table S3: Expression values and differential expression statistics of each gene. The result of DESeq2 differential expression test of all genes are indicated. Transcript: transcript ID, Sample_A: condition 1, Sample_B: condition 2, FDR: false discovery ratio calculated during DESeq2 analysis, Direction: up-regulated or down-regulated, or non-significant (NS), TPM_Mean_A: average expression value of condition 1, TPM_SD_A: standard deviation value of condition 1, TPM_Mean_B: average expression value of condition 2, TPM_SD_B: standard deviation value of condition 2, FC: fold change (TPM_Mean_B / TPM_Mean_A).

Table S4: Enriched gene ontology terms: Gene ontology terms enriched in between each condition. Rows 1-2: Conditions compared, Row 3: down-regulated or up-regulated genes, Row 4: gene ontology category (Biological process, Molecular function, Cellular component), Row 5: gene ontology term ID, Row 6: *p*-value calculated from GOStat, Row 7: the odds ratio value from GOStat, Row 8: the expected count value from GOStat, Row 9: the count value from GOStat, Row 9: the size value from GOStat, Row 10: name of gene ontology term.

**CRediT author statement**

**Yuki Yoshida:** Conceptualization; Data curation; Formal analysis; investigation; Methodology; Validation; Visualization; Roles/Writing - original draft; Writing - review & editing. **Daiki D. Horikawa:** Methodology. **Tetsuya Sakashita:** Methodology. **Yuichiro Yokota**: Methodology. **Yasuhiko Kobayashi:** Supervision. **Masaru Tomita:** Supervision; Resources. **Kazuharu Arakawa:** Conceptualization; Funding acquisition; Project administration; Resources; Writing - review & editing.

**Acknowledgments**

This work was funded by ImPACT, and partly by research funds from the Yamagata Prefectural Government and Tsuruoka City, Japan for KA and MT. YY was funded by Japan Society for the Promotion of Science (JSPS), grant number JP18J2115. The *Chlorella* used to feed tardigrades was gifted from Chlorella Industry.

**Declaration of Competing Interest**

The authors declare that they have no known competing financial interests or personal relationships which have or could be perceived to have influenced the work reported in this article.

**Ethics Statement**

All experiments were conducted following the Japanese law and guidelines from The Science Council of Japan, as well as the Ministry of Education, Culture, Sports, Science and Technology (MEXT) of Japan.

**References**

1	D.D. Horikawa, T. Kunieda, W. Abe, M. Watanabe, Y. Nakahara, F. Yukuhiro, T. Sakashita, N. Hamada, S. Wada, T. Funayama, C. Katagiri, Y. Kobayashi, S. Higashi and T. Okuda, Establishment of a rearing system of the extremotolerant tardigrade *Ramazzottius varieornatus:* a new model animal for astrobiology, Astrobiology. 8 (2008) 549-556. https://doi.org/10.1089/ast.2007.0139.

2	S. Andrews, FastQC a quality-control tool for high-throughput sequence data. (2015). http://www.bioinformatics.babraham.ac.uk/projects/fastqc (accessed May 21, 2015).

3	M.G. Grabherr, B.J. Haas, M. Yassour, J.Z. Levin, D.A. Thompson, I. Amit, X. Adiconis, L. Fan, R. Raychowdhury, Q. Zeng, Z. Chen, E. Mauceli, N. Hacohen, A. Gnirke, N. Rhind, F. di Palma, B.W. Birren, C. Nusbaum, K. Lindblad-Toh, N. Friedman and A. Regev, Full-length transcriptome assembly from RNA-Seq data without a reference genome, Nat. Biotechnol. 29 (2011) 644-652. https://doi.org/10.1038/nbt.1883.

4	Y. Yoshida, G. Koutsovoulos, D.R. Laetsch, L. Stevens, S. Kumar, D.D. Horikawa, K. Ishino, S. Komine, T. Kunieda, M. Tomita, M. Blaxter and K. Arakawa, Comparative genomics of the tardigrades *Hypsibius dujardini* and *Ramazzottius varieornatus*, PLoS Biol. 15 (2017) e2002266. https://doi.org/10.1371/journal.pbio.2002266.

5	M.I. Love, W. Huber and S. Anders, Moderated estimation of fold change and dispersion for RNA-seq data with DESeq2, Genome Biol. 15 (2014) 550. https://doi.org/10.1186/s13059-014-0550-8.

6	S. Falcon and R. Gentleman, Using GOstats to test gene lists for GO term association, Bioinformatics. 23 (2007) 257-258. https://doi.org/10.1093/bioinformatics/btl567.

7	K. Arakawa, K. Mori, K. Ikeda, T. Matsuzaki, Y. Kobayashi and M. Tomita, G-language Genome Analysis Environment: a workbench for nucleotide sequence data mining, Bioinformatics. 19 (2003) 305-306. https://doi.org/10.1093/bioinformatics/19.2.305.

8	K. Arakawa and M. Tomita, G-language system as a platform for large-scale analysis of high-throughput omics data, J. Pestic. Sci. 31 (2006) 282-288. https://doi.org/10.1584/jpestics.31.282.
