# Supplementary material for: RNA sequencing data for gamma radiation response in the extremotolerant tardigrade Ramazzottius varieornatus
Source: Data Brief. 2021 May 9;36:107111. doi: 10.1016/j.dib.2021.107111 (PMC8166744; doi:10.1016/j.dib.2021.107111)
Supplement: Supplementary file 2 [file mmc2.zip › mmc2/S2352340921003954.zip!/processed-artwork-DIB-D-21-00305-1619163921.zip!/images/extracted/DIB A1_Group0/DIB A1_Group0-cmyk.pdf]

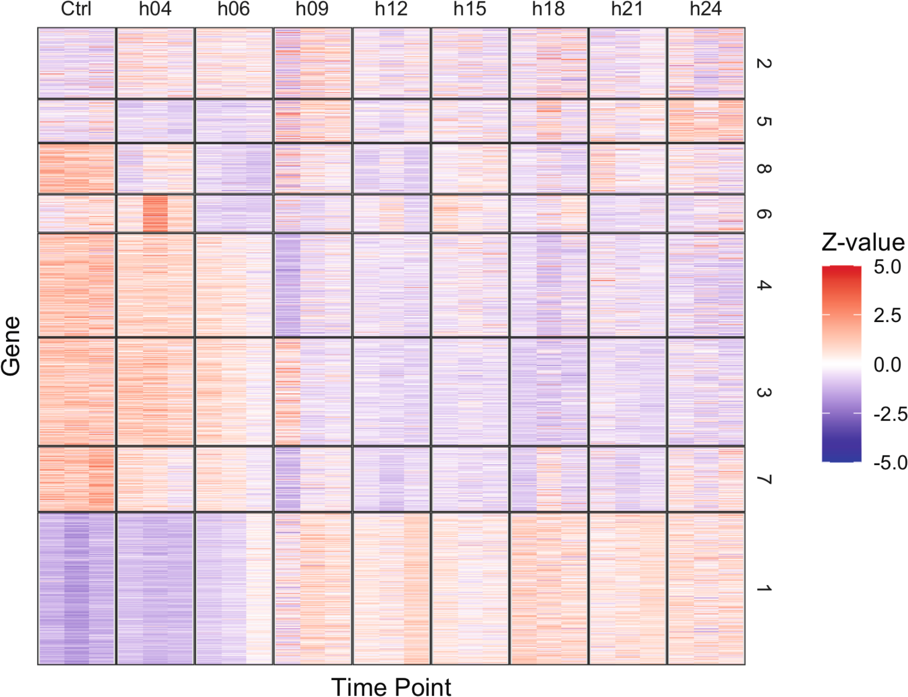

Ctrl
h04
h06
h09
h12
h15
h18
h21
h24
2
5
8
6
4
3
7
1
Gene
Time Point
Z-value
5.0
2.5
0.0
-2.5
-5.0
